# Predictors of Mortality of Streptococcal Bacteremia and the Role of Infectious Diseases Consultation: A Retrospective Cohort Study

**DOI:** 10.1093/cid/ciae168

**Published:** 2024-03-25

**Authors:** Nicolas Fourre, Virgile Zimmermann, Laurence Senn, Marion Aruanno, Benoit Guery, Matthaios Papadimitriou-Olivgeris

**Affiliations:** Infectious Diseases Service, Lausanne University Hospital, Lausanne, Switzerland; Infectious Diseases Service, Lausanne University Hospital, Lausanne, Switzerland; Infectious Diseases Service, Lausanne University Hospital, Lausanne, Switzerland; Infection Prevention and Control Unit, Lausanne University Hospital, Lausanne, Switzerland; Institute of Microbiology, Lausanne University Hospital, Lausanne, Switzerland; Infectious Diseases Service, Lausanne University Hospital, Lausanne, Switzerland; Infectious Diseases Service, Lausanne University Hospital, Lausanne, Switzerland; Infection Prevention and Control Unit, Lausanne University Hospital, Lausanne, Switzerland; Infectious Diseases Service, Cantonal Hospital of Sion and Institut Central des Hôpitaux (ICH), Sion, Switzerland

**Keywords:** streptococci, source control, infectious diseases consultation, sepsis, bloodstream infection

## Abstract

**Background:**

Streptococcal bacteremia is associated with high mortality. Thia study aims to identify predictors of mortality among patients with streptococcal bacteremia.

**Methods:**

This retrospective study was conducted at the Lausanne University Hospital, Switzerland, and included episodes of streptococcal bacteremia among adult patients from 2015 to 2023.

**Results:**

During the study period, 861 episodes of streptococcal bacteremia were included. The majority of episodes were categorized in the Mitis group (348 episodes; 40%), followed by the Pyogenic group (215; 25%). Endocarditis was the most common source of bacteremia (164; 19%). The overall 14-day mortality rate was 8% (65 episodes). The results from the Cox multivariable regression model showed that a Charlson comorbidity index >4 (*P* .001; hazard ratio [HR], 2.87; confidence interval [CI]: 1.58–5.22), *Streptococcus pyogenes* (*P* = .011; HR, 2.54;CI: 1.24–5.21), sepsis (*P* < .001; HR, 7.48; CI: 3.86–14.47), lower respiratory tract infection (*P* = .002; HR, 2.62; CI: 1.42–4.81), and absence of source control interventions within 48 hours despite being warranted (*P* = .002; HR, 2.62; CI: 1.43–4.80) were associated with 14-day mortality. Conversely, interventions performed within 48 hours of bacteremia onset, such as infectious diseases consultation (*P* < .001; HR, 0.29; CI: .17–.48) and appropriate antimicrobial treatment (*P* < .001; HR, .28; CI: .14–.57), were associated with improved outcome.

**Conclusions:**

Our findings underscore the pivotal role of infectious diseases consultation in guiding antimicrobial treatment and recommending source control interventions for patients with streptococcal bacteremia.

## Teaser

The overall 14-day mortality rate among 861 episodes of streptococcal bacteremia was 8%. Interventions performed within 48 hours of bacteremia onset, such as infectious diseases consultation, appropriate antimicrobial treatment, and source control were associated with improved outcome.

Streptococci, a diverse class of bacteria, are a frequent etiological agent of bacteremia, particularly in the case of community-acquired infections. This class encompasses more than 100 identified species, each categorized into distinct groups based on unique characteristics such as hemolysis type, antigens, and virulence factors [1]. These groups are associated with various types of infections, with *Streptococcus pneumoniae*, for instance, standing as a prominent cause of community-acquired pneumonia, whereas species within the Mitis and Bovis groups are commonly linked to infective endocarditis [2].

Streptococcal bacteremia is associated with increased morbidity and mortality, with several factors being associated with worse outcome, including advanced age, the presence of comorbidities, and the onset of sepsis [3–6]. Although the importance of promptly initiating appropriate antimicrobial treatment has been previously emphasized, the potential influence of infectious diseases consultation and early source control has remained relatively unexplored within the context of streptococcal bacteremia [7, 8].

This study aims to identify predictors of mortality in patients with streptococcal bacteremia and address this knowledge gap by focusing on the role of early management strategies, including infectious diseases consultation, the administration of appropriate antimicrobial treatment, and the consideration of source control.

## METHODS

This retrospective study was conducted at Lausanne University Hospital in Switzerland over an 8-year period spanning from 2015 to 2023. The study involved the merging of data from 2 distinct cohorts: the bacteremia cohort, which retrospectively included patients from January 2015 to December 2021, and the cohort of patients with suspected infective endocarditis, covering the period from January 2015 to June 2023 (retrospective inclusion: 2015–2017; prospective inclusion: 2018–2023). The ethics committee of the Canton of Vaud approved the study (CER-VD 2021-02516, CER-VD 2017-02137).

Inclusion criteria were adult patients (aged ≥18 years) and presence of at least 1 blood culture for *Streptococcus* spp. (database of the microbiology laboratory). Exclusion criteria consisted of patients who had formally declined the use of their data, cases with incomplete medical records (including patients transferred to other hospitals at the onset of infection without follow-up data), and instances where the isolated *Streptococcus* spp. was considered to be a contaminant.

Blood cultures were incubated using the BacT/ALERT System (bioMerieux, Marcy l'Etoile, France). Species identification was performed using matrix-assisted laser desorption-ionization time of flight mass spectrometry (Bruker Daltonics, Bremen, Germany) from 07:00 to 19:00. The various streptococcal species, with the exception of *S. pneumoniae*, were categorized into different groups, including Pyogenic, Mitis (apart from *S. pneumoniae*), Anginosus, Salivarius, Bovis, Sanguinis, and Mutans. Susceptibility results were obtained from the microbiology laboratory database and assessed in accordance with the European Committee on Antimicrobial Susceptibility Testing criteria [9].

The primary outcome of the study was the 14-day crude mortality rate. Data on demographics (age, sex), comorbidities, Charlson comorbidity index, laboratory results (white blood cell count, C-reactive protein levels) within 24 hours from first positive blood culture, antimicrobial treatment, source control, the presence of sepsis or septic shock, and the site of infection were retrieved from patients’ electronic health records. All data were collected, stored and managed using Research Electronic Data Capture (REDCap) by an infectious diseases specialist. REDCap electronic data capture tools is hosted at Lausanne University Hospital. REDCap is a secure, web-based software platform designed to support data capture for research studies [10, 11].

In our institution, infectious diseases consultants are informed of patients with positive blood cultures after species identification. In contrast to *Staphylococcus aureus* and *Candida* spp., infectious diseases consultation for streptococcal bacteremia is not mandatory [12, 13]. Follow-up blood cultures until sterilization were performed in patients exhibiting persistent symptoms or suspected of having infected endocarditis. Additionally, although not as common as in cases of *S. aureus* bacteremia or candidemia, clinicians often ordered follow-up blood cultures for patients with bacteremia involving any Gram-positive coccus.

The date of collection of the first positive blood culture was defined as bacteremia onset. A new episode was included if more than 30 days had passed since the cessation of antibiotic treatment for the initial bacteremia. The classification of bacteremia cases as community, healthcare-associated, or nosocomial followed the criteria established by Friedman et al [14] Sepsis or septic shock were defined in accordance with the criteria proposed by the Sepsis-3 International Consensus [15]. The diagnosis of infective endocarditis was made by the endocarditis team. The determination of the infection site was based on the assessment by the infectious diseases consultant, taking into account clinical, radiological, microbiological, and operative findings. Appropriate antimicrobial treatment was defined as the initiation of at least 1 antimicrobial agent with in vitro activity against the infecting isolate. Source control was considered warranted in the following situations: (1) removal of venous catheter in patients with catheter-related bacteremia or bacteremia of unknown origin with the presence of a venous catheter; (2) imaging-guided or surgical drainage of infected collections; (3) joint fluid drainage (arthrotomy, arthroscopy, needle aspiration); (4) cardiac surgery in endocarditis patients when indicated for heart failure; and (5) correction of urinary tract obstruction.

SPSS version 26.0 (SPSS, Chicago, IL, USA) was used for data analyses. Categorical variables were analyzed using the chi-square or Fisher exact test and continuous variables with Mann–Whitney *U* test. Univariate logistic regression models were assessed with 14-day mortality as the dependent variable. Clinically relevant noncollinear covariates, assessed through variance inflation factor, were used in multivariable analysis. After checking Cox assumptions, multivariable Cox proportional hazards regression models were performed with 14-day mortality as the time to event. Hazard ratios (HRs) and 95% confidence intervals (CIs) were calculated to evaluate the strength of any association. All statistic tests were 2-tailed and *P* < .05 was considered statistically significant. We finally performed Kaplan–Meier curves of the survival probability of patients with streptococcal bacteremia according to appropriate source control within 48 hours from bacteremia onset.

## RESULTS

**Figure 1.**
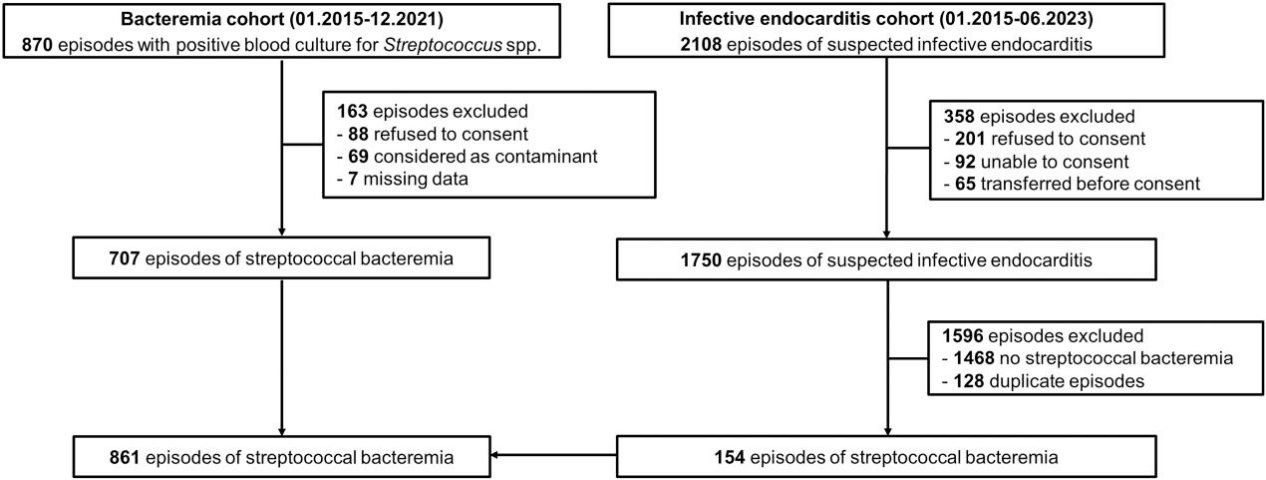
Flowchart of patients’ selection.

A total of 1152 episodes of streptococcal bacteremia were identified across both cohorts, with 861 involving 804 patients included (Figure 1). Within the Pyogenic group (215 episodes; 25%), *Streptococcus agalactiae* was the most frequently isolated species (94; 11%), followed by *Streptococcus dysgalactiae* (62; 7%), and *Streptococcus pyogenes* (59; 7%). The Mitis (apart from *S. pneumoniae*), Anginosus, Salivarius, Bovis, Sanguinis, and Mutans groups accounted for 348 (40%), 139 (16%), 78 (9%), 68 (8%), 12 (1%), and 9 (1%), respectively. *S. pneumoniae* was responsible for 36 cases (4%). Eighty-six (10%) isolates demonstrated resistance or susceptibility with increased exposure to penicillin.

Most bacteremias were linked to endocarditis (164; 19%) and abdominal infection (162; 19%), followed by bacteremia of unknown origin (158; 18%), skin and soft tissue (114; 13%), lower respiratory tract (86; 10%), and bone and joint infections (85; 10%). Follow-up blood cultures until sterilization were performed in 647 (75%) episodes; among them, only 4 (0.6%) had persistent bacteremia for at least 48 hours.

The overall 14-day mortality rate was 8% (65 episodes). *S. pyogenes* was the only species associated with 14-day mortality (Table 1). Sepsis occurred in 297 (34%) episodes. Infectious diseases consultation was provided in 680 (79%) cases; in 608 (71%) of these episodes, it was delivered within 48 hours from bacteremia onset. Antimicrobial treatment was initiated within 48 hours in 831 (97%) episodes and was considered appropriate in 829 (95%) episodes. Source control was deemed necessary in 320 (37%) episodes and was performed within 48 hours of bacteremia onset in 199 (62%) (Supplementary Table 1). Time to blood culture positivity of each streptococcal group was not associated with 14-day mortality.

**Table 1.** Comparison of Survivors and Nonsurvivors

	Survivors (n = 796)		Nonsurvivors (n = 65)		*P*
Demographics					
Male sex	540	68%	46	71%	.680
Age (y)	65	50–76	74	65–85	<.001
Age >60 y	485	61%	52	80%	<.001
Co-morbidities					
Diabetes mellitus	174	22%	15	23%	.876
Obesity (body mass index ≥30 kg/m^2^)	178	24%	6	9%	.011
Chronic kidney disease (moderate or severe)	102	13%	12	19%	.252
Malignancy (solid organ or hematologic)	200	25%	28	43%	.003
Immunosuppression^a^	200	25%	14	22%	.654
Chronic obstructive pulmonary disease	81	10%	9	14%	.396
Cirrhosis	56	7%	5	8%	.801
Congestive heart failure	59	7%	12	19%	.007
Charlson comorbidity index	4	2–6	7	5–9	<.001
Charlson comorbidity index >4	384	48%	50	77%	<.001
Setting of bacteremia onset					
Community	447	56%	33	51%	
Healthcare-associated	94	12%	11	17%	
Nosocomial	255	32%	21	32%	1.000
Microbiological data					
Two or more blood cultures positive (initial blood cultures)	462	58%	40	62%	.604
Pathogens					
*Streptococcus pneumoniae*	31	4%	5	8%	.183
Pyogenic group	192	24%	23	35%	.052
*Streptococcus pyogenes*	49	6%	10	15%	.010
* Streptococcus agalactiae*	88	11%	6	9%	.836
* Streptococcus dysgalactiae*	55	7%	7	11%	.220
Bovis group	62	8%	6	9%	.633
Mitis group^a^	326	41%	22	34%	.294
Sanguinis group	12	2%	0	0%	1.000
Anginosus group	130	16%	9	14%	.727
Salivarius group	72	9%	6	9%	1.000
Mutans group	9	1%	0	0%	1.000
Polymicrobial bloodstream infection	201	25%	28	43%	.003
Time to positivity (h)					
*S. pneumoniae* (n = 36 episodes)	10	8–12	8	8–10	.282
Pyogenic group (n = 199 episodes)	9	7–11	8	6–10	.165
*S. pyogenes* (n = 55 episodes)	10	7–12	8	6–12	.539
* S. agalactiae* (n = 89 episodes)	9	7–11	7	5–9	.074
* S. dysgalactiae* (n = 55 episodes)	10	7–11	8	7–10	.786
Bovis group (n = 61 episodes)	10	8–12	11	10–14	.362
Viridans streptococci (n = 511 episodes)	13	11–18	14	10–19	.815
Mitis group^b^ (n = 329 episodes)	13	10–16	14	9–17	.731
Anginosus group (n = 132 episodes)	18	14–25	19	15–22	.762
Salivarius group (n = 74 episodes)	12	10–13	10	8–18	.711
Increased exposure or resistant to penicillin	81	10%	5	8%	.668
Persistent bacteremia (≥48 h)	4	.5%	0	0%	1.000
Infection data					
Fever	699	88%	47	72%	.002
Sepsis	243	31%	54	83%	.001
Septic shock	85	11%	35	54%	<.001
Laboratory data within 24 h from first positive blood culture					
White blood cells (×10^9^/L)	10	6–14	12	5–19	.047
Neutropenia	112	14%	3	5%	.035
C-reactive protein (mg/L) (n = 711 patients)	110	54–202	161	89–263	.002
Type of infection					
Unknown origin	148	19%	10	15%	.691
Catheter-related	20	3%	2	3%	.679
Endocarditis	154	19%	10	15%	.513
Lower respiratory tract infection	67	8%	19	29%	<.001
Abdominal infection	151	19%	11	17%	.869
Skin and soft tissue infection	111	14%	3	5%	.034
Bone and joint infection	78	10%	7	11%	.828
Other focus	105	13%	8	12%	1.000
Management					
Infectious diseases consultation	652	82%	28	43%	<.001
Infectious diseases consultation within 48 h	584	73%	24	37%	<.001
Source control					
Not warranted	503	63%	38	59%	
Warranted and performed within 48 h	189	24%	10	15%	
Warranted, but not performed within 48 h	104	13%	17	26%	.009
Antimicrobial initiation within 48 h	775	97%	56	86%	<.001
Appropriate antimicrobial within 48 h	766	96%	54	83%	<.001

Data are depicted as number and percentage or median and Q1–3.

^a^Ongoing immunosuppressive treatment at bacteremia onset, intravenous chemotherapy in the 30 d before bacteremia onset, acquired immunodeficiency syndrome, neutropenia, and asplenia.

^b^Without *S. pneumoniae.*

The results from the Cox multivariable regression model (Table 2) showed that a Charlson comorbidity index >4 (*P* = .001; HR, 2.87; CI: 1.58–5.22), *S. pyogenes* (*P* = .011; HR, 2.54; CI: 1.24–5.21), sepsis (*P* < .001; HR, 7.48; CI: 3.86–14.47), lower respiratory tract infection (*P* = .002; HR, 2.62; CI: 1.42–4.81), and absence of source control interventions within 48 hours despite being warranted (*P* = .002; HR, 2.62; CI: 1.43–4.80) were associated with 14-day mortality. Conversely, interventions performed within 48 hours of bacteremia onset, such as infectious diseases consultation (*P* < .001; HR, 0.29; CI: .17–.48) and appropriate antimicrobial treatment (*P* < .001; HR, 0.28; CI: .14–.57) were associated with improved outcome.

**Table 2.** Cox Proportional Hazard Multivariable Regression of 14-day Mortality Among Patients With Streptococcal bacteremia

	*P*	HR (95% CI)
Charlson comorbidity index >4	.001	2.87 (1.58–5.22)
*Streptococcus pyogenes*	.011	2.54 (1.24–5.21)
Infectious diseases consultation within 48 h	<.001	.29 (.17–.48)
Sepsis	<.001	7.48 (3.86–14.47)
Lower respiratory tract infection	.002	2.62 (1.42–4.81)
Appropriate antimicrobial treatment within 48 h	<.001	.28 (.14–.57)
Source control		
Not warranted	Reference	
Warranted and performed within 48 h	.861	1.07 (.52–2.21)
Warranted, but not performed within 48 h	.002	2.62 (1.43–4.80)

Abbreviations: CI, confidence interval; HR, hazard ratio.

**Figure 2.**
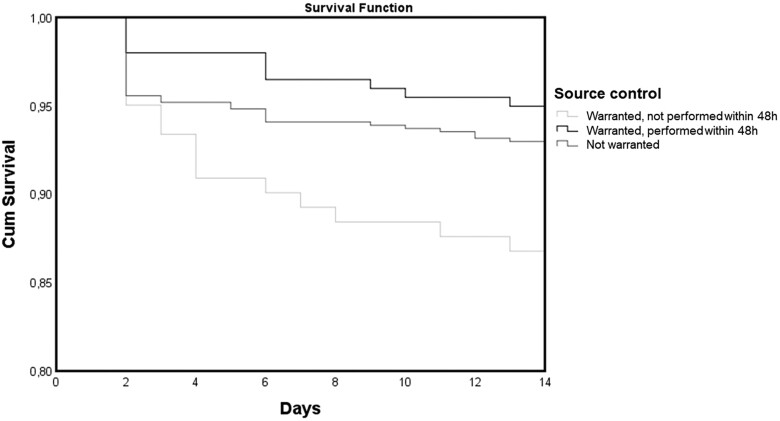
Kaplan–Meier curves of the survival probability of patients with streptococcal bacteremia according to need and performance of early source control.

Figure 2 shows Kaplan–Meier survival probability curves for episodes with streptococcal bacteremia based on the need for and performance of early source control. Early source control was linked to a more favorable outcome when compared to episodes where it was warranted but not performed (*P* = .004).

Recurrence of bacteremia from the same streptococcal species within 1 year of the initial episode was observed in 19 (2%) episodes. No difference was observed among episodes with or without infectious diseases consultation within 48 hours from bacteremia onset (2% vs 2%; *P* = 1.000).

## DISCUSSION

In this study, we examined the factors influencing mortality in patients with streptococcal bacteremia and underscored the critical importance of infectious diseases consultation and early management with appropriate antimicrobial treatment, and timely source control when needed.

The 14-day mortality rate was 8%, which falls within the range typically documented in the literature [3–6, 16–18]. Notably, *S. pyogenes* exhibited the highest mortality rate among the different streptococcal species (17%), also comparable to previous findings [3]. One plausible explanation for this heightened mortality associated with *S. pyogenes* is its frequent association with sepsis (51% vs 33%; *P* = .010). Additionally, *S. pyogenes* is renowned for producing various virulence factors and its connection to toxic shock syndrome [4].

As expected, a higher Charlson comorbidity index was independently associated with worse outcomes [3–6, 17]. A prior meta-analysis on invasive pneumococcal diseases supported these findings, showing that both components of the Charlson comorbidity index (age and comorbidities) were linked to increased mortality [17]. Additionally, as observed in the present study, the same meta-analysis unveiled that lower respiratory tract infections and sepsis were linked to worse outcomes [17]. Both factors were previously shown to be associated with increased mortality in patients with bacteremia [4, 18, 19].

Our study underscores the necessity of a comprehensive approach to managing streptococcal bacteremia. Early interventions, such as infectious diseases consultation, tailored antimicrobial treatment, and source control when indicated play pivotal roles in enhancing patient outcomes.

Notably, although the positive impact of infectious diseases consultation on the management and outcomes of patients with *S. aureus* bacteremia and candidemia has been previously established, its influence in streptococcal bacteremia had not been thoroughly examined [12, 13]. In a previous study involving patients with *S. pyogenes* bacteremia, infectious diseases consultation did not directly affect outcomes, but it did lead to more tailored antibiotic treatment, including the deescalation to narrow-spectrum beta-lactam antibiotics and the avoidance of combination therapy with clindamycin in nonsevere cases [8]. To the best of our knowledge, our study is the first to demonstrate that infectious diseases consultation within 48 hours from bacteremia onset was associated with improved outcomes among patients with streptococcal bacteremia. Although a bedside consultation for streptococcal bacteremia is not mandatory in our institution, a majority of patients (71%) received infectious diseases consultation within the first 48 hours from bacteremia onset, resulting in improved management characterized by more appropriate antibiotic treatment and higher rates of early source control when warranted.

The absence of early source control and the administration of inappropriate antimicrobials emerged as independent predictors of mortality in our study. Although the significance of promptly initiating appropriate antimicrobial treatment in improving survival among patients with streptococcal bacteremia has been previously underscored, the role of early source control had not been explored in prior research [7]. Notably, in patients with necrotizing fasciitis, the majority of which is instigated by streptococci, the timing of surgical debridement has been shown to impact prognosis, with those receiving early surgical treatment experiencing lower mortality rates [20].

The time to positivity of blood cultures for various pathogens has previously been associated with mortality, particularly in the context of *S. aureus* bacteremia. However, among different groups of streptococci, conflicting results have been reported [6, 16, 21, 22]. In our current study, we did not find any association between the time to positivity of any streptococcal group and mortality. The time to positivity in blood cultures typically serves as an indicator of the microbial load of the infecting organism. However, variations in results among different studies may be attributed to discrepancies in blood culture collection protocols and the duration between blood culture collection and processing in the incubation machine in different centers. These factors introduce variability into the results and impact the interpretation of the time to positivity in various studies.

Nonetheless, our study has its limitations. First, it is a retrospective single-center study. Given that our center is a tertiary university facility, the cohort examined represents the most complex cases and may not be fully representative of the epidemiology in nontertiary centers. Additionally, there are numerous confounding factors that can influence the association between improved survival and early interventions, such as the decision to limit or withdraw care and the willingness of surgeons or interventional radiologists to perform source control interventions. In our study, only 4 patients died within 48 hours from bacteremia onset, with an additional 2 experiencing a withdrawal of care in the same time frame, thus having limited impact on our findings. Furthermore, although we considered the appropriateness of empirical antimicrobial treatment, we did not assess the specific types of antimicrobials, duration, or eventual oral switch. Moreover, hospitalization length and readmission were also not included.

In conclusion, this study endeavors to bridge this knowledge gap and provide insights into the multifaceted approach required for the effective management of streptococcal bacteremia. Our findings emphasize the crucial role of infectious diseases consultation in guiding antimicrobial treatment and recommending source control interventions for patients with streptococcal bacteremia, thereby contributing to the enhancement of patient care and the reduction of mortality.

## Supplementary Data

Supplementary materials are available at *Clinical Infectious Diseases* online. Consisting of data provided by the authors to benefit the reader, the posted materials are not copyedited and are the sole responsibility of the authors, so questions or comments should be addressed to the corresponding author.

## Supplementary Material

ciae168_Supplementary_Data

## Notes

***Author Contributions*.** M. P. O. and B. G. conceived the idea. N. F., V. Z., L. S., and M. A. collected the patients’ data. B. G. supervised the project. M. P. O. performed the analysis and interpreted the results. M. P. O. wrote the manuscript. All authors contributed to manuscript revision and read and approved the submitted version.
