# Supplementary material for: Predictors of Mortality of Streptococcal Bacteremia and the Role of Infectious Diseases Consultation: A Retrospective Cohort Study
Source: Clin Infect Dis. 2024 Mar 25;78(6):1544–50. doi: 10.1093/cid/ciae168 (PMC11175677; doi:10.1093/cid/ciae168)
Supplement: ciae168_Supplementary_Data [file ciae168_supplementary_data.pdf]

Supplementary Table 1. Source control procedures warranted and performed depending of infection site

Infection site	Source control warranted	Type of source control procedure	Early source control performed
Unknown origin (n=158)	58 (37%)	Removal of central or peripheral venous catheter	41 (71%)
Catheter-related (n=22)	22 (100%)	Removal of central or peripheral venous catheter	17 (77%)
Endocarditis[a] (n=164)	56 (34%)	Valvular replacement or removal of CIED	20 (36%)
Lower-respiratory tract (n=86)	7 (8%)	Drainage of empyema	5 (71%)
Abdominal (n=162)	70 (43%)	Drainage of abscess or peritonitis, correction of biliary-tract obstruction	44 (63%)
Skin and soft tissue (n=114)	27 (24%)	Drainage of abscess	19 (70%)
Bone or joint (n=85)	56 (66%)	Drainage of joint fluid or abscess, drainage or replacement of osteoarticular prosthetic material	28 (50%)
Other[b] (n=113)	48 (42%)	Replacement of vascular prothesis, treatment of mycotic aneurysm, correction of urinary-tract obstruction	35 (73%)

Data are depicted as number (percentage)

[a]including cardiac implantable electronic device lead infection
